# Micro-Chemical Researches on the Digestion of Starch and Amylaceous Foods

**Published:** 1856-04

**Authors:** Philip Burnard Ayres

**Affiliations:** London.


### ARTICLE XVI.

"*Micro-chemical Researches on the Digestion of Starch and Amylaceous Foods.*" By Philip Burnard Ayres, M. D., London. Communicated by John Bishop, Esq., F. R. S. Received January 11, 1855.

After some general historical remarks on the methods hitherto employed in the investigation of the complicated phenomena of the process of digestion, the comparatively small results obtained by chemical analysis of the contents of the stomach, intestinal canal, and of the evacuations, by Tiedmann and Gmelin, Berzelius, and others, the author proceeded to demonstrate the necessity of a minute examination of the contents of the alimentary canal by the microscope, and such chemical tests as we possess for the determination of the changes of such articles of food as exhibit definite structure.

In order that we may ultimately arrive at a complete exposition of the phenomena of digestion, he is of the opinion that it will be necessary to examine—first, the structure of particular kinds of food, then the changes produced in them by cooking, and lastly to trace the changes they undergo at short intervals, through the alimentary canal from the stomach to the rectum. The results of a series of researches of this character on the changes in starch, and starch containing foods, are presented in this memoir.

The method adopted for the examination of the changes in starch and starch-foods was as follows: an animal was kept fasting twenty-four hours, and afterwards confined to a diet consisting of the starch or amylaceous food, with water, for five or six days, until the debris of all other kinds of food previously taken were cleared from the alimentary canal. At a determinate time, after a meal, the animal was killed, the abdomen laid open as quickly as possible, and ligatures placed at short intervals on the intestinal canal, from the pyrolus to the rectum. The contents of the stomach and each portion of the intestinal canal included between the ligatures was then carefully examined. This mode of examination sufficed to determine the changes which occur in the food during normal digestion; but other questions as to the particular secretion or secretions by which the changes observed were effected.

The fluids poured into the alimentary canal are five in number—the saliva, gastric juice, bile, pancreatic juice, and finally, the intestinal mucus.

The influence of the saliva is easily determined, by chewing the particular food subjected to experiment, and keeping the mixture at about 98° Fahr. The combined action of the saliva and gastric juice is seen in the contents of the stomach. To determine the action of the bile, the common bile-duct was tied, and to ascertain the action of the intestinal mucus, it was necessary to ligature the bile and pancreatic ducts. If the digestion of the substance is not effected in the stomach, it is evident that it cannot be attributed to the saliva or gastrice juice; if the digestion is still effected in the intestinal canal after ligature of the bile-duct, it cannot be attributed to the action of the saliva, gastric juice or bile; if it still go on after ligature of the bile and pancreatic ducts, the digestive power must of necessity be referred to the action of the intestinal mucus, provided no change has previously taken place in the stomach; but if the food passes unchanged after cutting off the supply of bile and pancreate juice, but proceeds after ligature of the bile-duct alone, the act of digestion must be referred to the pancreatic juice.

The author first briefly describes the structure of the starches and starch-containing vegetables employed in his experiments; then the changes produced by cooking, and finally enters on a minute description of the changes observed in the experiments he performed on normal digestion, and after cutting off the supply of bile and pancreatic juice.

The correct appreciation of the structure of the starch-granule is of considerable importance in relation to these investigations, and the author believes that he has been able to afford a satisfactory solution of this vexed question. The changes observed during the digestion of starch favor the original opinion of Leuwenhoeck, that the starch-granule consists essentially of an investing membrane or cell-wall, enclosing an amorphous matter, the true starch, which strikes an intense blue color with iodine; and these changes also support the opinion of Professor Quekett, that the concentric circles seen on the starch-granules of many plants are simple foldings of the investing membrane, leaving it still doubtful, however, whether these concentric circles are not in the starches of some plants composed of linear series of dotted elevations or depressions of the investigating membrane.

By these experiments it was determined that the concentric circles remain after the whole of the starch matter, colorable by iodine, was removed, and that even then the characteristic cross and colors were still seen when the granules were viewed by polarized light, although more feebly than before; this result being probably due to the lessened power of refracting light, after the removal of the starch matter.

After describing the structure of the wheat-grain and flour, the changes occurring in the wheat-starch during the manufacture of bread are given in detail; but the most interesting of the changes produced by cooking are those seen in the boiled or roasted potato and in the boiled pea.

In each of these the act of cooking effects two purposes: it causes great enlargement and physical change of the starch-granules, and dissolves the intimate adhesion of the starch-cells, which afterwards appear as ovid or globular, slightly adherent bodies distended by the swollen starch granules, the outlines of which are indicated by more or less irregular gyrate lines, produced by the mutual compression of the starch-granules within an inelastic cell-membrane.

The starch-granules of the pea possess a much thicker investing membrane than those of the potato, which causes their outlines to remain much more distinct after the removal of the true starch substance during theprocess of digestion. The other structures seen in the pea are carefully described; the most curious among them being the cells composing the external layer of the testa, which bear so strong a resemblance to columnar epithelium of the intestine, that they might be mistaken for the latter by an inattentive observer.

The substances submitted to experiment were—1, boiled wheat-starch; 2, wheaten bread; 3, uncooked tous les mois; 4, boiled tous les mois; 5, boiled potato; 6, uncooked peas; 7, boiled peas; 8, boiled peas after ligature of the bile-duct; 9, boiled potatoes after ligature of the bile and pancreatic ducts. Several subsidiary experiments were made to determine the action of the intestinal mucus, the saliva, and the substance of the pancreas, on starch.

The conclusions at which the author arrives from the experiments are:

1. That the starch-granule is composed of two parts, chemically and histologically distinct, a cell-membrane and homogeneous contents. The markings seen on many varieties of starch are referred to folds or markings of the investing membrane.

2. No perceptible change occurs in the starch, whether raw or cooked, during its sojourn in the stomach of quadrupeds or the ventriculus succenturiatus and gizzard of birds; all the granules preserve their perfect reaction with iodine and their pristine appearance.

3. The conversion of boiled starch into dextrine and glucose is chiefly effected in the first few inches of the small intestine, but it continues to take place in a less degree throughout the entire intestinal canal.

4. In the digestion of boiled wheat or other starch, or of wheaten bread, the bulk of the mass rapidly diminishes in its passage through the small and large intestines, so that it ultimately yields only a small quantity of fæcal matter. After being deprived of their contents, the membranes of the granules shrink and shrivel up into a minute granular matter, which constitutes the chief bulk of the fæcal evacuations after an exclusive diet of starch food.

5. The digestion of raw starch food (peas) in the pigeon or other granivorous birds goes on much more slowly, and progresses pretty equally throughout the entire intestinal canal. The starch-granules, whether free or included in cells, become intersected by radiating or irregular lines or fissures, more or less opaque or granular; they also gradually lose their characteristic reaction with iodine; and this important change, commencing at the surface, progresses towards the centre, until the whole of the starch matter is removed, leaving the starch-membranes often apparently whole, retaining their characteristic markings. The fissured and granular condition of the starch-granules is not due to their trituration in the gizzard, but to the action of the intestinal fluids, since it was often seen in granules enclosed in and protected by perfect starch-cells. In the digestion of raw starch food, a considerable quantity always escapes change, for many starch-cells and granules in the fæces perfectly retain the characteristic reaction with iodine.

6. As the starch remains unchanged in the stomach, its conversion into glucose cannot be attributed to the saliva or gastric juice, unless we suppose these fluids to remain inactive in the stomach, and suddenly to regain their activity in the first part of the small intestine. The author found that the saliva was capable of effecting the conversion of starch into glucose, but that the mixture of saliva and gastric juice in the stomach did not possess that property even after being rendered alkaline by carbonate of soda. It is probable that the converting power of the saliva, as it flows from the mouth, depends not on the true saliva, but on the buccal mucus; for Magendie found that saliva taken from the parotid duct was wholly inactive, while the mixed saliva from the mouth effected the conversion with great facility. Unless, then, the sublingual and submaxillary glands secrete a different fluid from the parotids, it is evident that the activity of the saliva must be attributed to the buccal mucus.

7. The difference between the digestion of boiled and raw starch in dogs is seen in the experiments on the digestion of boiled wheat-starch, boiled tous les mois, and bread. In all these, some starch-granules escape the action of heat and water, and remain in nearly their pristine condition. These uncooked starch-granules undergo slow and imperfect changes, being fissured, broken, and more or less altered, but, in general, retaining their characteristic reaction with iodine.

8. The conversion of starch into glucose is not effected by the bile, since after ligature of the common bile-duct, the changes occur to as great an extent as when the bile passes freely into the intestinal canal.

9. It is not due to the pancreatic juice, inasmuch as after ligature of the bile and pancreatic ducts in the same animal, the digestion of starch is still effected.

10. The only remaining secretion is the intestinal mucus, which is especially abundant at the upper part of the intestinal canal; and a further proof is afforded of the activity of the intestinal mucus taken from the upper part of the duodenum above the entrance of the pancreatic duct after ligature of this duct and the common bile-duct, by its capability of converting a large quantity of fresh-boiled starch into glucose out of the body.

11. In the cooking of starch containing vegetables, such as potatoes and peas, the adhesion of the starch-cells is dissolved or weakened so as to render them easily separable and amenable to the action of the intestinal fluids. At the same time the starch-granules undergo a large increase in bulk, distend the cells, and by their mutual compression, their outlines present the appearance of gyrate lines beneath the cell-wall. The cells seldom burst so as to admit their contents, or present any appreciable opening through which the intestinal fluids can directly penetrate. The author cannot positively affirm so much of the starch-membranes, because these are so extremely delicate that fissures might be invisible, but he believes that in a great number the membranes remain entire.

12. If this be the case, the conversion of starch matter into glucose must be effected by the permeation or endosmose of the intestinal fluids through the invisible pores of two membranes, in the digestion of the pea, the potato, and other similar foods, and the glucose must escape through the same membranes by exosmose.

13. Before the conversion of starch into glucose, the amylaceous matter contained in the starch is more dense than the intestinal mucus in immediate contact with the cells, and an inward current or endosmose is established; but after that conversion the syrupy fluid is less dense than the mucus, and then an outward current or exosmose occurs, by which the glucose escapes from the cells into the intestine and is absorbed. If this be the case, as the details of the experiments tend strongly to prove, a new and important function is assigned to the intestinal mucus.

14. In normal dsgestion, chyme escapes very slowly from the stomach into the duodenum, in small quantities, as it is detached from the alimentary mass by the muscular movements of the stomach, and this gradual propulsion often occupies several hours after a meal. This slow propulsion is evidently intended to expose the comminuted food fully to the action of the intestinal juices, and produce an intimate mixture with them. The comparatively empty condition of the upper part of the small intestine, even during active digestion, is thus fully explained.

15. If the food be too finely divided or incapable of a second solidification in the stomach, it passes too rapidly into the first part of the small intestine, is insufficiently mixed with the intestinal fluids, and a considerable part escapes digestion. On the other hand, if it enters the small intestine in masses incapable of reduction by the muscular action of the parts or solution in the fluid, it traverses the intestinal canal unchanged, except at the surface, which is then alone exposed to the action of the intestinal fluids.

16. It is not necessary for the conversion of starch into glucose that the fluids in the duodenum or other parts of the intestinal canal should be alkaline, or even neutral, for in several of the experiments the contents of every part of the alimentary canal had an acid reaction.

17. The greater part of the intestinal mucus is not excrementitious, for little, if any, mucus is perceptible in the fæces in normal digestion, except at their surface, whereas the greater proportion of the contents of the small intestine consists of mucus. A considerable quantity of mucus is seen in the cæcum, but it rapidly diminishes in the colon, and is scarcely detectible in the fæces, except that on the surface, which is probably derived from the mucous membrane of the rectum. The author raises the question, whether one of the chief functions of the cæcum is not to effect the conversion of the intestinal mucus into some other substance capable of re-entering the blood, and performing some ulterior purpose in the animal economy.

18. In normal digestion, the separation of the epithelium of the mucous membrane of the intestine is the exception instead of the rule, as stated by some physiologists. The author questions the theory of the detachment of the epithelium of the villi in each act of absorption, on the grounds that the presence of detached epithelium was unfrequent in the whole course of his experiments; that epithelium is readily detached by manipulation; that the continual reproduction of such a vast amount of cell-tissue must necessarily be accompanied by a vast expenditure of vital force; and finally, that it is not necessary, because fluids readily penetrate epithelial membranes.

19. The passage of a given food through the whole length of the intestinal canal may occupy a comparatively short time, especially when the animal is fasting. In one experiment, where a pigeon refused food until the fæces contained no visible debris of previous food, starch-granules were detected in the fæces within two hours after a meal, and this although the intestine of this animal is extremely narrow, and about a yard in length.

20. A remarkable circumstance in the digestion of starch or starch foods is the constant presence of myriads of vibriones in the lower part of the intestinal canal. They are generally first observed in the lower part of the small intestine, as minute brilliant points, just visible with a power of 600 diameters, in active movement. They increase in numbers towards the cæcum, in which a large number of fully-developed vibriones are constantly seen. These minute organisms increase in size and length in the colon and rectum, and their fissiparous mode of propagation, first described by the author in the "Quarterly Journal of Microscopical Science," may be distinctly traced by examining the contents of these portions of the intestine.

